# Proactively Adjusting Stopping: Response Inhibition is Faster when Stopping Occurs Frequently

**DOI:** 10.5334/joc.264

**Published:** 2023-05-04

**Authors:** Roos A. Doekemeijer, Anneleen Dewulf, Frederick Verbruggen, C. Nico Boehler

**Affiliations:** 1 Ghent University, Ghent, Belgium

**Keywords:** proactive inhibitory control, response inhibition, proactive adjustments, SSRT, stop-trial frequency

## Abstract

People are able to stop actions before they are executed, and proactively slow down the speed of going in line with their expectations of needing to stop. Such slowing generally increases the probability that stopping will be successful. Surprisingly though, no study has clearly demonstrated that the speed of stopping (measured as the stop-signal reaction time, SSRT) is reduced by such proactive adjustments. In addition to a number of studies showing non-significant effects, the only study that initially had observed a clear effect in this direction found that it was artifactually driven by a confounding variable (specifically, by context-independence violations, which jeopardize the validity of the SSRT estimation). Here, we tested in two well-powered and well-controlled experiments whether the SSRT is shorter when stopping is anticipated. In each experiment, we used a Stop-Signal Task, in which the stop-trial frequency was either high (50%) or low (20%). Our results robustly show that the SSRT was shorter when stop signals were more anticipated (i.e., in the high-frequent condition) while carefully controlling for context-independence violations. Hence, our study is first to demonstrate a clear proactive benefit on the speed of stopping, in line with an ability to emphasize going or stopping, by trading off the speed of both.

## Introduction

To achieve long-term goals, we need to be able to stop more immediate but unwanted or obsolete actions and impulses (response inhibition). For instance, response inhibition is critical when a pedestrian needs to stop themselves from stepping onto the street after a reckless driver unexpectedly runs a red light. In this scenario, response inhibition is largely *reactive*, i.e., the success depends on the in-the-moment ability to stop (Verbruggen, McLaren, & Chambers, 2014; Wessel, 2018). When the need for stopping can be expected on the other hand (e.g., in case drivers run that particular red light on a daily basis), the success of response inhibition also depends on the ability to *proactively* adjust settings. In fact, it has been argued that the success of inhibition is largely dependent on proactive inhibitory control rather than on reactive control when stopping can be anticipated, as is the case in typical lab settings (Wang, 2013; Wessel, 2018).

**Figure 1:**
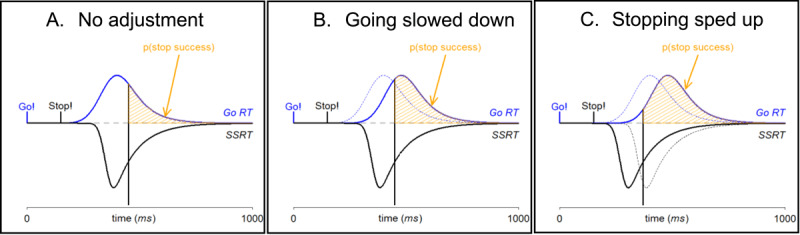
The race between go and stop and the various ways in which proactive control could improve the success of response inhibition, as indicated by the probability of stopping, p(stop success): **(A)** base scenario with no proactive adjustments, **(B)** the effect of slowing down the go response (i.e., the Go RT distribution shifts to the right), and **(C)** the additional effect of speeding up the stop response (the SSRT distribution shifts to the left). Note that other factors, such as the onset of the go stimulus (Go!) or the delay of the stop signal (Stop!), remained unchanged.

In the popular Stop-Signal Task for example, participants need to execute a simple go task as fast and accurately as possible, unless an infrequent stop signal appears after a delay (the stop-signal delay, SSD); if such a stop signal appears, participants need to inhibit their already-initiated go response (Logan & Cowan, 1984). Response inhibition in this task has been described as a horse race between a go response and a stop response, running to execute and inhibit a response respectively. The success of inhibition depends on the relative speed of the go response (which can be measured as the overt reaction time to the go stimulus, or Go RT) versus the speed of the stop response (the stop-signal reaction time, SSRT; which cannot be overtly measured, but can be estimated under the assumptions of the horse-race model; Logan & Cowan, 1984; see Figure 1A). Participants typically slow down their go responses if stop signals can occur, which is commonly referred to as proactive slowing (Verbruggen & Logan, 2009a; Vink, Kaldewaij, Zandbelt, Pas, & Plessis, 2015). The adjustment furthermore happens in line with the frequency at which stop signals occur: slowing is more pronounced when stop signals appear relatively often (Bissett & Logan, 2011; Jahfari et al., 2012; Logan, 1981; Leunissen, Coxon, & Swinnen, 2016; Logan & Burkell, 1986; Messel, Raud, Hoff, Skaftnes, & Huster, 2019; Messel, Raud, Hoff, Stubberud, & Huster, 2021; Ramautar, Kok, & Ridderinkhof, 2004; Verbruggen & Logan, 2009a). These findings suggest that participants proactively slow down their go response in anticipation of possibly needing to stop.

Importantly, such proactive slowing of the go response by itself, and all else being equal, improves the likelihood that inhibition will be successful even if the latency of the stop response is not influenced at all (see Figure 1B). Still, while proactive slowing through its effects on going might affect response inhibition only indirectly (i.e., the outcome of the race is ‘rigged’ in favor of the stop response by slowing down the go response), neuroimaging studies have shown that also the processes involved in stopping are adjusted when participants expect stop signals to occur (for a review see Aron, 2011). For example, Ramautar et al. (2004) manipulated the stop-trial frequency in an EEG study, and found that the amplitude and latency of the stop-P3 (i.e., an ERP component related to the stop response; Wessel & Aron, 2015) were larger and earlier when stop-trial frequency was high (50%) compared to low (20%). Furthermore, both Chikazoe et al. (2009) and Jahfari et al. (2012) used cues to indicate whether a stop signal was likely to appear or not, and showed that inhibition-related brain activity (defined as brain activity that associated with the stopping after a signal has been presented) was lower, and preparation-related brain activity (brain activity associated with the anticipation of a stop signal) higher, when stopping was anticipated. Combined, these findings suggest that stopping seems to be facilitated in line with the extent that going is slowed down; this suggests that people strike a balance between going and stopping, and that this balance is shifted according to anticipations of needing to stop (Chikazoe et al., 2009; which is also supported by TMS research, see Jahfari, Stinear, Claffey, Verbruggen, & Aron, 2010).

Correspondingly, it can be expected that the speed of stopping (i.e., measured as the SSRT) is faster, the more stopping is anticipated (see Figure 1C). Yet, the vast majority of previous work has only found numerical evidence in favor of this idea (see Table 1; both in trial-level cuing and block-level manipulation contexts). Given the theoretical motivation for such an effect, stop-related adjustments shown with fMRI and EEG, and the consistent observations of numerical trends in the expected direction, we consider that the nonsignificant results could simply be due to study-specific limitations. One recurring limitation, for example, is that sample size was rather low in most studies. Another concern about some of the previous studies is that the method that was used to estimate SSRT may not have been fully suited for the design at hand; specifically, many methods become inaccurate when proactive slowing is involved and are thus not suited to estimate the SSRT when stop-trial frequency is high (Verbruggen, Chambers, & Logan, 2013).

**Table 1:** List of previous studies that have used stop-trial frequency to examine effects of proactive control on SSRT, plus their main findings.


STUDY		SAMPLE SIZE	MANIPULATION		MEAN SSRT DIFFERENCE		RESULTS	
			
*AUTHORS*	*YEAR*		*HIGH*	*LOW*	*REPORTED*	*EFFECT SIZE* ^3^	*TEST*	*P*

Logan & Burkell	1986	12	80%, 50% versus 20%		plotted	*NA*	*F*(2,44) = 1.34, *MS_e_* = 4789	Insig.

Ramautar et al.	2004	14	50%	20%	plotted	*NA*	*F*(1,13) = 0.01	0.91

Ramautar et al.	2006	16	50%	20%	–10 msec	–0.25	*F*-test	Insig.

Verbruggen & Logan (Exp. 5)	2009a	18	70%	30%	–13 msec	-1.1	*F* < 1	Insig.

Bissett & Logan	2011	24	40%	20%	–19 msec	*NA*	*F*(1, 23) = 3.07, *MS_e_* = 1395	<0.10; *B* = 1.58

Jahfari et al.	2012	16	50%	25%	–19 msec	-0.41	*F*-test	Insig.

Leunissen et al.	2016	22	40%	20%	–1 msec	-0.03	*t* = 0.17	0.869

Messel et al.	2019	28	66%	25%	–24 msec	-0.45	*z* = –1.62	0.106

Bissett et al.	2021	136^1^	40%	20%	–16 msec	*NA*	*ANOVA*	<0.001

Bissett et al.	2021	88^2^	40%	20%	–4 msec	*NA*	*ANOVA*	0.24

Messel et al.	2021	22	66%	25%	–20 msec	-0.39	*z*-test	0.354


* B = Bayes Factor. ^1^ Participants who had short SSDs on average, i.e., mean(SSD) < 300 msec. ^2^ Participants who had long SSDs on average, i.e., mean(SSD) > 300 msec. ^3^ Computed by us as Hedge’s *g*: i.e., the mean SSRT difference divided by the mean standard deviations (i.e., Cohen’s *d*_av_; Lakens, 2013) multiplied by an approximation of Hedges’ correction factor (see Goulet-Pelletier & Cousineau, 2018). *NA* indicate that the effect size could not be computed due to missing information.

Interestingly, Bissett, Jones, Poldrack, and Logan (2021) recently reanalyzed data of 522 participants and indeed initially found the expected result that the SSRT was shorter when stopping could be anticipated (i.e., when the stop-trial frequency was high, 40%, versus low, 20%). However, they argued that their results could have been inaccurate because one of the key assumptions of the horse-race model was severely violated (i.e., the assumption of context independence, which refers to the idea that the go response is unaffected by the stop signal). Upon accounting for the data of the participants with severe violations, the effect of stop-trial frequency on SSRT was eliminated. Consequentially, there have still been no studies so far that have shown the expected effect that stopping is faster when anticipated. Furthermore, the recent work by Bissett et al. (2021) highlights the need to check for possible context-independence violations in such research.

**Figure 2:**
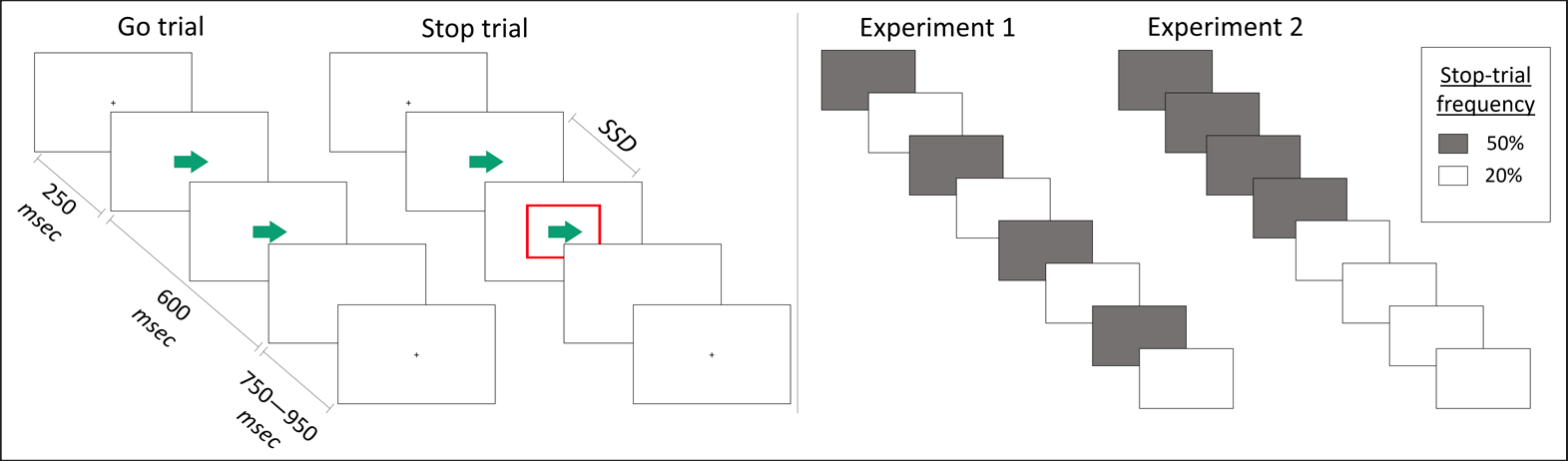
Overview of experiment set-ups for Experiment 1 and Experiment 2. (left) The Stop-Signal Task that we used in both experiments; the stop trials were randomly interleaved with go trials and comprised either 50% (high-frequent condition) or 20% (low-frequent condition) of the trials within a block. (right) The sequence of high- and low-frequent blocks; the starting block was counterbalanced between participants.

In the present study, we aimed to clarify whether the SSRT is shorter when the need to stop is anticipated, while also checking for signs of context-independence violations that could confound such results. We conducted two well-powered experiments, in which we manipulated the stop-trial frequency (high: 50% versus low: 20%) in a typical Stop-Signal Task (see Figure 2). Both experiments were designed fully in accordance to the latest recommendations concerning task implementation and analysis of Stop-Signal Task data (Verbruggen et al., 2019), with the exception of having blocks with a high stop-trial frequency (which is typically discouraged to limit proactive slowing), since this was the objective of our study here. Nevertheless, we took some additional measures to account for proactive slowing (i.e., in the participant selection, feedback messages, and data analysis; see methods section) and conducted various checks to establish any violations of the context-independence assumption, in order to ensure the accuracy of our SSRT estimates. By doing so, we established that the SSRT is indeed shorter when stopping is anticipated, which importantly took place in the absence of signs of context-independence violations.

## Methods

### Participants

For the first experiment (Experiment 1), we recruited 50 Psychology Bachelor students of Ghent University via an online platform (SONA) to participate in the experiment online. Nine participants were excluded according to predefined (and previously used, see Doekemeijer, Verbruggen, & Boehler, 2021) criteria: (1) incomplete data (2 participants excluded); (2) the experiment not being the main focus on the web browser at any time during the experiment (4 participants); (3) poor performance on go trials (go accuracy < 90%; 3 participants); (4) poor performance on stop trials (*p(respond|signal)* < 30% or > 70%, more on this below; 0 participants); (5) evidence for a violation of the context-independence assumption (average RT on failed stop-trials > average Go RT on go trials; 0 participants); and (6) slowing over the course of the experiment (Go RT in the first block < 1.5 times the Go RT in the last block of each condition; 0 participants). So, the final sample consisted of 41 participants: 39 female and two male, aged 19 (*SD* = 3.17) on average; meaning that our final sample had a rather severe gender skew.

For the second experiment (Experiment 2), we followed the same procedure as in Experiment 1 and recruited 64 participants. A total of 18 were excluded according to the above criteria: (1) incomplete data (8 participants); (2) the experiment not being the main focus on the browser at any time during the experiment (2 participants); (3) poor performance on go trials (5 participants); (4) poor performance on stop trials (3 participants); (5) evidence for a violation of the context-independence assumption (0 participants); and (6) slowing over the course of the experiment (0 participants). The final sample consisted of 46 participants, of which 42 female and four male, again showing a gender skew. The average age of the sample was 19 (2.23).

Using G*Power (Faul, Erdfelder, Lang, & Buchner, 2007), we conducted sensitivity analyses to determine the effect sizes that we would be able to detect for a (two-tailed) paired *t*-test with *α* = *β* = 0.05 and our above-mentioned sample sizes. For Experiment 1 (*n* = 41) and Experiment 2 (*n* = 46), we would be able to detect effects of size 0.58 and 0.54, respectively.

### Task and procedure

We used the same Stop-Signal Task with visual go and stop signals (programmed in JsPsych, De Leeuw, 2015) in both experiments; both experiments were conducted online (hosted on the Ghent University staff webspace); the research was conducted according to the ethical rules presented in the General Ethical Protocol of the Faculty of Psychology and Educational Sciences of Ghent University. On go trials, participants needed to indicate whether an arrow (the go stimulus) was pointing left or right, using the left or right arrow-key respectively; on stop trials, an additional stop signal appeared as a red box around the go stimulus after a variable delay (stop-signal delay, SSD). Each trial started with a fixation cross (shown for 250 msec), followed by the go stimulus (shown for 600 msec), the stop-signal after the SSD (on stop trials only; shown after the SSD until the start of the blank space), and a blank space (that had a variable duration between 750–950 msec) (see left section of Figure 2 for a visual depiction). The SSD started at 200 msec and was subsequently updated after each stop trial according to an adaptive staircase procedure: if participants failed to inhibit their response on a stop trial, the SSD decreased by 50 msec (down to a minimum of 50 msec); if participants were successful, the SSD increased by 50 msec (up to a maximum of 550 msec). This procedure has been shown to result in about a 50% probability to respond on a stop trial, *p(respond|signal)*, which is optimal for estimating SSRT (Band, Van der Molen, & Logan, 2003). Furthermore, since the tracking adjusts to the participant’s performance, it may automatically accounts for (some) proactive slowing. Importantly, for the tracking to work properly, participants were instructed to respond as fast and accurately as possible to both go trials and stop trials, and were discouraged from waiting for the stop signal to occur.

The experiments consisted of eight experimental blocks of 160 trials each, in which we manipulated the stop-trial frequency on a block level: in four blocks the stop signal appeared on 50% of the trials (“high-frequent” blocks), while in the other four blocks the stop signal appeared on 20% of the trials (“low-frequent” blocks). Participants were informed at the start of each block on the frequency of the stop signals. In Experiment 1, the blocks alternated between high- and low-frequent; in Experiment 2, we clustered the low- and high-frequent blocks together, creating one stretch of low-frequent blocks and one of high-frequent blocks (each consisting of four blocks; see right section of Figure 2). The starting block/cluster of blocks was counterbalanced across participants, and the participants were aware of this manipulation.

A practice block preceded the experimental blocks. The practice block consisted of two phases: in the first phase, participants received instructions on the go trials and subsequently practiced with 8 go trials; in the second phase, participants received instructions about the stop trials and then practiced with 40 trials. Notably, in Experiment 1, the stop-trial frequency in all practice stop trials was 20%, so the participants did not practice with any high-frequent blocks. Since we had clusters of blocks in Experiment 2, we changed this procedure slightly there: participants practiced with a short block (20 trials) with 20% stop-trial frequency before starting the cluster of low-frequent experimental blocks and practiced with a short block with 50% stop-trial frequency before the high-frequent experimental blocks.

After the practice block(s) and experimental blocks, participants received a feedback screen containing their average reaction time on go trials, the amount of go trials that they had not responded to (‘go omissions’), and the *p(respond|signal)* of the current and previous block of the same block type. The feedback screen also contained warnings if (1) they had slowed down more than 10% compared to their previous average reaction time of the same condition, (2) they had more than 2% go omissions or (3) they responded to 30% or fewer stop trials (which implies that the participants were waiting excessively on the stop signal to occur), and where reminded to not wait for the stop signal to occur.

### Analysis plan

All analyses were conducted in R version 4.0.3 (R Core Team, 2014); all data and analyses scripts are available on OSF: https://osf.io/vfm8b/. We first investigated the go performance in the two conditions. In line with previous work, we expected that the mean reaction time on go trials (mean Go RT) and the amount of choice errors (i.e., pressing the wrong button) would be lower in the high-frequent blocks than in the low-frequent blocks. We additionally expected the rate of go omissions (i.e., non-responses on no-signal trials) to be higher in the high-frequent condition than in the low-frequent condition (Verbruggen & Logan, 2009a), despite the fact that this was clearly discouraged by instruction. We conducted paired *t*-tests to test these hypotheses (using base R) and established the corresponding effect size (Cohen’s *d*; using the ‘rstatix’ R package, Kassambara, 2021).

We investigated the effect of proactive control on the speed of stopping by estimating SSRTs using the integration approach with replacement of go omissions (Verbruggen et al., 2019). In this approach, SSRTs are estimated from the Go RTs, the SSDs, and the overall probability to execute the go response while a stop signal was present, *p(respond|signal)* (Logan & Cowan, 1984; Verbruggen et al., 2019). Specifically, Go RTs are ranked from short to long and the *n*th Go RT is determined, in which *n* equals the participant’s *p(respond|signal)* times the number of no-signal trials. Subsequently, the mean SSRT is calculated as the *n*th Go RT minus the average SSD. In the specific version we used, go omissions are replaced by long Go RTs (here: 1250 msec) before ranking them. This version has been shown to be most accurate among typical SSRT estimation approaches, and to be invariant to the shape of the Go RT distributions (Verbruggen et al., 2019). Still, as an extra safeguard against a possible confounding influence of slowing continuously over the course of the experiment, we ran an additional analysis, in which we explicitly accounted for continuous slowing by estimating the SSRT per block, and subsequently averaging them to obtain an unbiased SSRT (as done before by Verbruggen, et al., 2013).

Note that we additionally fit a more novel, parametric model to our data in an exploratory analysis to estimate SSRTs, while accounting for trigger failures (i.e., instances where the stop response was not triggered by the stop signal). However, the model did not adequately capture our (high-frequent) data in Experiment 1. We hypothesized that this may be due to either (1) the feedback procedure, in which participants got feedback on their performance directly before switching to another block type; resulting in feedback that may not immediately be relevant, given that participants likely approached the two types of task blocks somewhat differently (e.g., differential degrees of proactive slowing); (2) the fact that participants never practiced with the high-frequent condition (because training was exclusively done with the more typical low-frequent condition); and/or (3) that the previous block type may have had carry-over effects into the block of the other block type (e.g., which was supported by our finding that the difference between low- and high-frequency blocks was smaller at the very beginning of a block than towards the end of a block, see Figure A in the Appendix). In Experiment 2, we solved these issues in the design: we had consecutive blocks of the same block types, and a practice phase preceding both experimental phases at the respective stop-trial probability. However, when modeling the resulting data, the misfit remained in Experiment 2, suggesting that these factors did not drive the factor underlying this misfit. We further report our findings and our (unsuccessful) attempts to solve the misfit in the supplementary materials on OSF.

## Results

### Go performance

An overview of the performance on go trials is provided in Table 2. Here, slowing was defined as the difference between mean Go RT in the first block minus the mean Go RT in the last block of the same condition. Overall, there was negligible across-block slowing within conditions and experiments.

**Table 2:** Per experiment, an overview of the go and stop performances for high- versus low-frequent conditions, and the results of the corresponding paired t-test if applicable. Standard deviations are listed inside the parentheses.


	EXPERIMENT 1 (*n* = 41)					EXPERIMENT 2 (*n* = 46)				
	
	HIGH	LOW	*t*(40)	*p*	*dz*	HIGH	LOW	*t*(45)	*p*	*d_Z_*

**Go trials**											

Slowing	31 (110)	18 (56)	–	–	–	7 (67)	18 (40)	–	–	–

Go RT	500 (81)	427 (61)	10.25	<0.001	1.60	521 (112)	430 (70)	9.54	<0.001	1.41

Go omissions	2.9 (3.0)	0.8 (1.4)	4.70	<0.001	0.73	2.6 (2.7)	0.7 (0.9)	4.88	<0.001	0.72

Choice errors	1.3 (1.3)	2.1 (1.8)	–4.11	<0.001	–0.64	1.2 (1.2)	2.0 (1.8)	–3.90	<0.001	–0.58

**Stop trials**										

SSD	260 (83)	185 (65)	–	–	–	275 (97)	182 (74)	–	–	–

p(respond|signal)	49.1 (4.1)	51.3 (3.1)	–	–	–	48.7 (5.0)	51.1 (2.1)	–	–	–

SRRT	434 (56)	389 (50)	9.28	<0.001	1.45	458 (81)	395 (65)	11.07	<0.001	1.63

SSRT	224 (38)	235 (41)	–3.40	0.002	–0.53	231 (24)	243 (25)	–3.17	0.003	–0.47

SSRT per block	226 (32)	237 (39)	–3.21	0.003	–0.50	231 (24)	244 (25)	–3.39	0.001	–0.50


*d_z_* = Cohen’s *d*; SSD = stop-signal delay; SRRT = signal-respond reaction time; SSRT = stop-signal reaction time as estimated using the integration approach with replacement of go omissions; SSRT per block = averaged SSRT calculated per block (i.e., to account for proactive slowing). *Note*: Accuracy (i.e., go omissions, choice errors; and p(respond|signal)) is reported in percentages; latency (all other variables) is in msec.

As expected, we found that the Go RT was significantly and consistently longer in the high-frequent condition than the low-frequent condition. We also found that participants made significantly more go omissions in the high-frequent condition than the low-frequent condition, in line with Verbruggen and Logan (2009a). We additionally found that participants made fewer choice errors in the high-frequent than low-frequent condition. Overall, these findings correspond to previous reports showing that people proactively adjust their go response in anticipation of stop signals (Bissett & Logan, 2011; Elchlepp, Lavric, Chambers, & Verbruggen, 2016; Leunissen, et al., 2016; Logan, 1981; Logan & Burkell, 1986; Ramautar et al., 2004) and in particular, that people respond more cautiously (Jahfari et al., 2012; Verbruggen & Logan, 2009a).

### Stop performance

Again, the overview of the stop performance can be found in Table 2. In addition, we have plotted the overall participants’ probabilities to stop as a function of SSD (i.e., the “inhibition function”) and SSD distributions in the Appendix (see Figure B; individual plots can be found on OSF).

Before estimating the SSRT, we checked whether our data would provide accurate estimates. We established that the *p(respond|signal)* had adequately converged to 50%, as was expected from the adaptive staircase procedure. Furthermore, we checked for any context-independence violations in four ways. First, we checked whether the average reaction time on failed stop trials (signal-respond RT, or SRRT) was numerically lower than that of the Go RT (which was the case, see Table 2). Second, we checked whether the mean SRRT increased progressively over SSD. Third, we plotted the cumulative density function of the Go RT on go trials, SRRTs on stop trials with short SSDs (i.e., all values below the mean SSD), and SRRTs on stop trials with long SSDs (all values over the mean SSD); and established whether these functions had a common minimum but different slopes in order of Go RT (least steep), SRRTs on long-SSD trials (steeper), and SRRT on short-SSD trials (steepest) (Verbruggen & Logan, 2009b). Lastly, we applied the check developed and used by Bissett et al. (2021), with which they showed that context independence was violated in their investigation on the effect of stop-trial frequency on SSRT. This check entails computing the differences between SRRT and a matching Go RT (i.e., the RT of the closest, accurate go trial that preceded the failed stop trial); positive values would indicate context-independence violations. Ultimately, we established that none of the four checks indicated that the context independence assumption was violated^1^ (see Figure C and Figure D in the Appendix for the last three checks for Experiment 1 and Experiment 2, respectively).

Since our data seemed to allow for accurate SSRT estimation, we applied the integration approach with replacement of go omissions per condition and experiment. We found that the mean SSRTs were shorter in the high-frequent blocks than the low-frequent ones (see Table 1). In order to further safeguard against any possible confounding influence of continuous slowing over the course of the experiment (and possibly more so for the high-frequent condition), in a subsequent analysis, we applied another method that estimates the SSRTs per block (again using the integration method with replacement), and subsequently averages them (Verbruggen, et al., 2013). We again found that the mean SSRTs were lower in the high-frequent blocks than the low-frequent blocks.

In an additional analysis suggested by a reviewer, we checked whether our effect could be attributed to (a build-up of) reactive adjustments to previous trials (i.e., whether the effect may be explained by sequential effects, as suggested earlier by Bissett & Logan, 2011; evidence of such buildups in our data can be seen in Figure A in the Appendix) rather than (or in addition to) the proactive adjustments made by the participants. Specifically, we accounted for sequential effects of the directly preceding trials by estimating the SSRT on exclusively go and stop trials that had been preceded by a go trial; we still found a significant difference in SSRT; Experiment 1: SSRT_high_ = 222 (SD = 36) msec, SSRT_low_ = 235 (43) msec; t (40) = –2.76, p < 0.05; Experiment 2: SSRT_high_ = 226 (24) msec, SSRT_low_ = 242 (25) msec; t (45) = –4.03, p < 0.001.

So, our findings robustly indicated that participants have a shorter SSRT when stop trials are more common, and that this effect was neither driven by confounding effects of proactive slowing nor by violations of the context-independence assumption of the horse-race model underlying SSRT estimation nor sequential effects.

## Discussion

In this paper, we investigated the effect of proactive control on the speed of stopping (estimated as the SSRT), which we examined by manipulating the stop-trial frequency (high: 50% versus low: 20%) in two experiments employing a typical Stop-Signal Task. We expected to find that the speed of the stop response would be faster when stopping could be anticipated (i.e., in the 50% condition), despite this not having been convincingly demonstrated yet. Following the latest recommendations with respect to experiment set-up and SSRT estimations (see Verbruggen et al., 2019), we established in two experiments that people indeed proactively adjusted their stop response in line with the frequency of the stop signal, and thus in line with their anticipations. Specifically, they displayed shorter SSRTs when stop-trials are more frequent. This study is therefore the first to clearly demonstrate that there is an effect of stop-trial frequency on the speed of the stop response, and thereby resolves the perceived discrepancy between the neuroimaging studies that show that stopping is affected by such anticipation on the one hand (e.g., Chikazoe et al., 2009; Ramautar et al., 2004, 2006) and behavioral work that showed insignificant results on the other (see Table 1).

Together with the well-known finding that people slow down their go responses in anticipation of needing to stop (which we also observed here), our main finding that stopping is sped up reinforces the idea that people balance the demands of the go response and stop response and that this balance is adjusted proactively (Chikazoe et al., 2009; Jahfari, et al., 2010; Verbruggen & Logan, 2009a; in addition to reactively adjusting within a given block, Strayer & Kramer, 1994, which we also briefly explored in Figure A in the Appendix). Although adjustments to the go response alone can already (to some extent) explain why stopping is improved at a behavioral level (as in Figure 1B), we here show that the latency of the stop response is also adjusted (Figure 1C), indicating that proactive control improves inhibition in two separable ways.

Still an important but unanswered question concerns *how* the stop response is affected proactively. For one, it is unclear at which stage(s) the stop response is improved, i.e., at the attentional, selection, and/or execution stage (Verbruggen et al., 2014). Another unanswered question concerns the role of trigger failures in the effect of proactive inhibitory control on the stop response. Trigger failures are instances where the stop response was not initiated, meaning that inhibition was doomed to fail. These failures have recently received ample attention in the response-inhibition field, initially because they can lead to incorrect conclusions about the SSRT if they are ignored (i.e., SSRTs are overestimated if trigger failures are ignored; Matzke, Love, & Heathcote, 2017). Additionally, trigger failures have been found to primarily explain various inter-individual response inhibition differences (e.g., people with ADHD have more trigger failures than healthy controls; Weigard, Heathcote, Matzke, & Huang-Pollock, 2019) as well as a prominent intra-individual difference (i.e., the rate of trigger failures is lower when stopping is rewarded compared to unrewarded; Doekemeijer, Verbruggen, & Boehler, 2021). In line with this emerging view that trigger failures are an important feature of stop performance, it seems plausible that proactive control primarily modulates the rate of trigger failures rather than (or in addition to) the SSRT. We attempted to answer this question here by applying a novel parametric model that is able to dissociate trigger failures versus SSRT, but were unsuccessful in doing so given that the model did not capture our high-frequent data well (see OSF for more detail: https://osf.io/vfm8b/?view_only=1bd83355355745e1a5a00e90b64599d5). This means that the results of this additional analysis are, at this point, inconclusive. So, our work clearly showed that there is an effect of proactive control on the stop response, but more work is needed to validly delineate how proactive control affects the stop response exactly.

More generally, our finding that the stop response is proactively adjusted was expected from the (numerical) trend-level effects on the SSRT that had been reported in previous studies. Most studies had insignificant results, presumably due to limited sample sizes^2^ and potentially by other factors, like the method that is used to estimate the SSRTs, for which the recommendations have evolved over recent years (Verbruggen et al., 2013; Verbruggen et al., 2019). A pertinent and recent exception is the study of Bisset et al. (2021). In that study, the authors initially established significant SSRT differences in a data set where stop-trial frequency had been manipulated (high: 40% versus low: 20%), but also found that the effect seemed to be caused by context-independence violations that happen when the stop signal follows the go stimulus very rapidly (i.e., at a short SSD). After accounting for that by excluding participants accordingly, the stop-trial probability effect on SSRT in their study disappeared, leading the authors to conclude that it was an artifact of the context-independence violations observed. Interestingly, in our study, we conducted four checks, including the one described by Bissett et al. (2021), and found no evidence that the assumption of context independence was violated in our sample. Possibly, the difference in findings can be attributed to differences in the complexity of the go task: we used simple stimulus-response mappings (i.e. arrow images that correspond to the arrow-key responses), whereas Bissett et al. (2021) used rather complex mappings (multiple shapes that correspond to arbitrary responses). This idea is further supported by a finding of Sánchez-Carmona et al. (2021) who used a simple go task and similarly to the present study did not find any evidence for context-independence violations. More research is however needed to structurally investigate whether (and if so, why) stimulus-response mappings may indeed relate to context-independence violations, which is an important venue to explore given Bissett et al. (2021)’s conclusion that such violations could critically undermine a large portion of the Stop-Signal Task literature. In this sense, our findings are reassuring in that context-independence violations are thus not ubiquitous in Stop-Signal Task data.

One limitation of our study, however, is that the integration method used to estimate the SSRT assumes that the stop response plays out equally on all trials, and thus that the SSRT is independent of SSD (i.e., the assumption of “stop context-independence”). In contrast to the violation of context-dependence described above, we note that the task designs used in our study potentially allows for violations of this stop context-independence assumption. Specifically, the upper bound of the SSD staircase was very close to the end of each trial (i.e., 50 msec before the end of the trial), so it is likely that the stop stimulus was gone before the stop response could run to full completion when SSDs were long. Although perception is not perfectly correlated with the exact duration of stimulation (e.g., Hawkins & Shulman, 1979), it is possible that the SSRT could therefore be prolonged on stop trials with particularly long SSDs (which were more prevalent on high-frequent blocks), meaning that violations of stop context-independence may be present in our (particularly high-frequent) data. However, it is unlikely that those potential stop context-independence violations confounded our finding that stopping is faster when anticipated, as the violations predict SSRT to be longer on high-frequent blocks, which is the opposite pattern of what we found here.

In summary, response inhibition can be proactively adjusted according to one’s anticipations of needing to stop. This proactive adjustment happens both by modulating the go response (i.e., by responding more cautiously) but also by modulating the stop response. Here, we particularly found evidence that the stop response is sped up (i.e., the SSRT is shorter) when stopping is anticipated (as estimated by two established variants of the non-parametric integration approach). Moreover, given the discrepancy between our results and those of Bissett et al. (2021) on the topic of context-independence violations of the horse-race model (which jeopardize the validity of the SSRT estimates) occur, more research is needed on establishing under what circumstances they arise.

## Data Accessibility statement

All code and data can be found on OSF: https://osf.io/vfm8b/.

## Additional File

The additional file for this article can be found as follows:

10.5334/joc.264.s1 Appendix. Figures A to D.

## Footnotes

Although we thus did not find consistent signs of (strong) context-independence violations in our data, we did find two patterns in our Figures that were inconsistent with the predictions of the horse-race model. We further elaborate on these inconsistences in Figure C and D of the Appendix.

Based on a reviewer’s suggestion, we have conducted an ad-hoc meta-analysis (using the ‘meta’ package in R, Balduzzi, Rücker, & Schwarzer, 2019); see supplementary analysis on OSF), which showed that the previous studies pooled effect size (Hedge’s *g*) was –0.41 (i.e., a medium effect size, as we also found in our studies). This provides further evidence that the insignificant trend-level findings may indeed have been due to limited sample sizes.
