# Supplementary material for: Proactively Adjusting Stopping: Response Inhibition is Faster when Stopping Occurs Frequently
Source: J Cogn. 2023 May 4;6(1):22. doi: 10.5334/joc.264 (PMC10162359; doi:10.5334/joc.264)
Supplement: Appendix. — Figures A to D. [file joc-6-1-264-s1.pdf]

Appendix

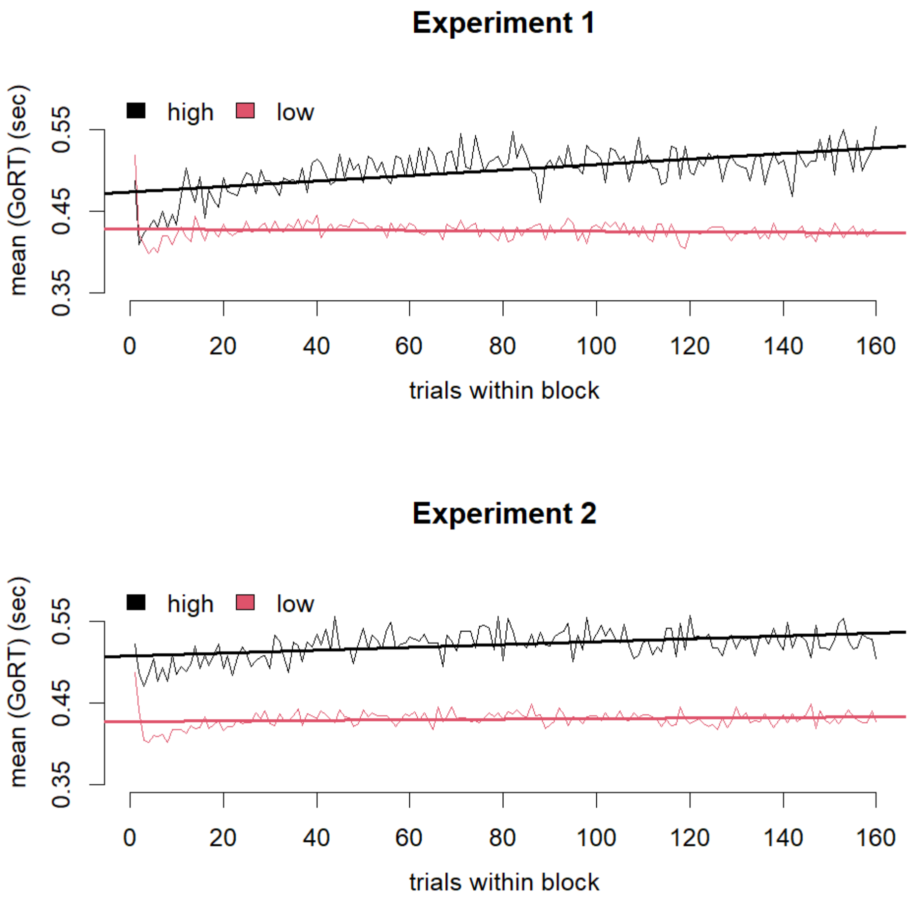

Figure A. *Progression of Go RT on go trials of Experiment 1 (top) and Experiment 2 (bottom) averaged over trials within the blocks of each block type (high- versus low-frequent blocks), depicting that (1) Go RTs in high-frequent blocks are already higher than low-frequent blocks at the start of each block, indicating that participants adjusted their performance based on instructions (i.e., there was already proactive adjustment); and that (2) the difference in Go RT between block types increases as the trials increase, suggesting that there were additionally sequential effects at play (i.e., (a buildup of) reactive adjustments) within the block types. Note that we still found that the SSRT was lower in the high-frequent blocks than in the low-frequent blocks even when accounting for the sequential effects.*

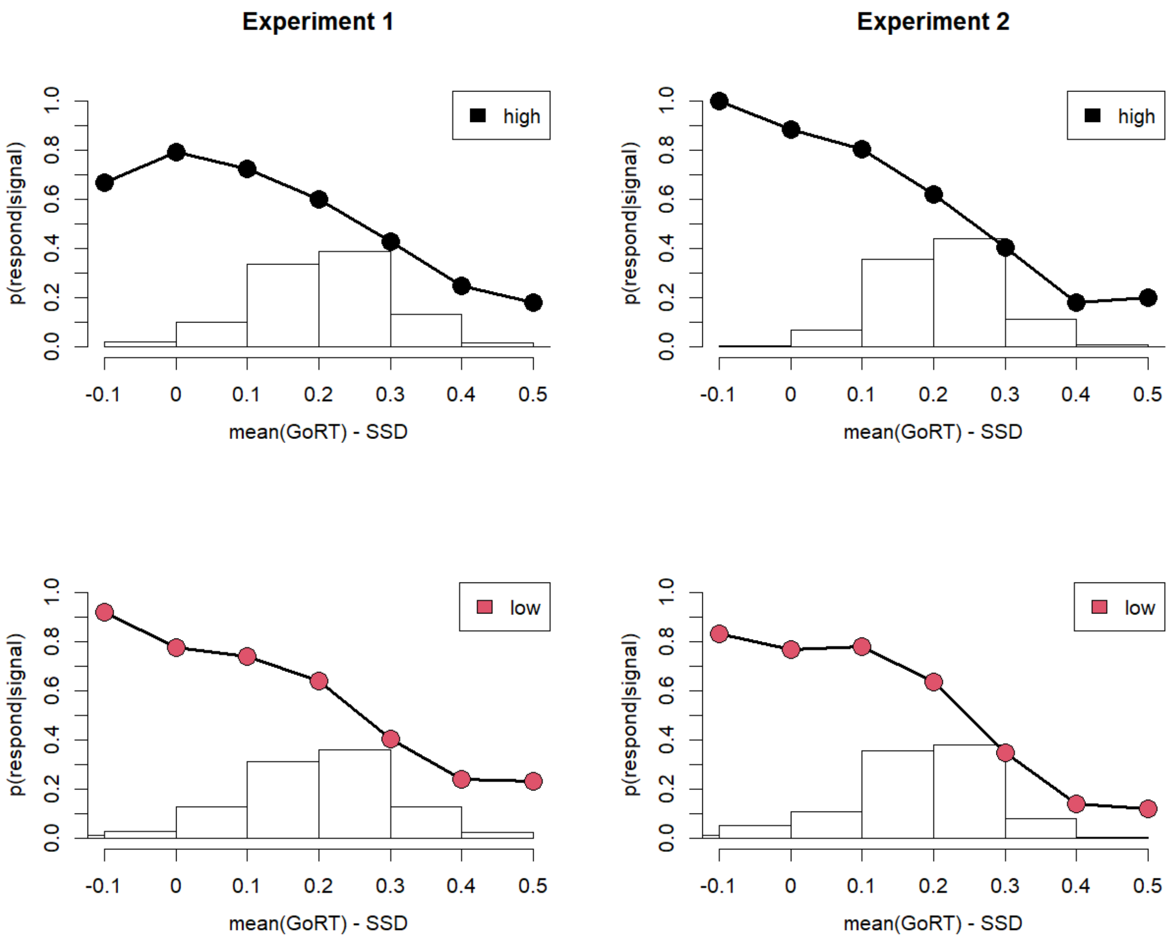

Figure B. *Overview of aggregated stop performance (SSD distribution, plotted on the x-axes; and inhibition functions) of participants in Experiment 1 (left) and Experiment 2 (right). Both the distribution and the inhibition function show SSDs that have been adjusted so that the plots align over participants by subtracting the SSD from the mean (Go RT) of each participant.*

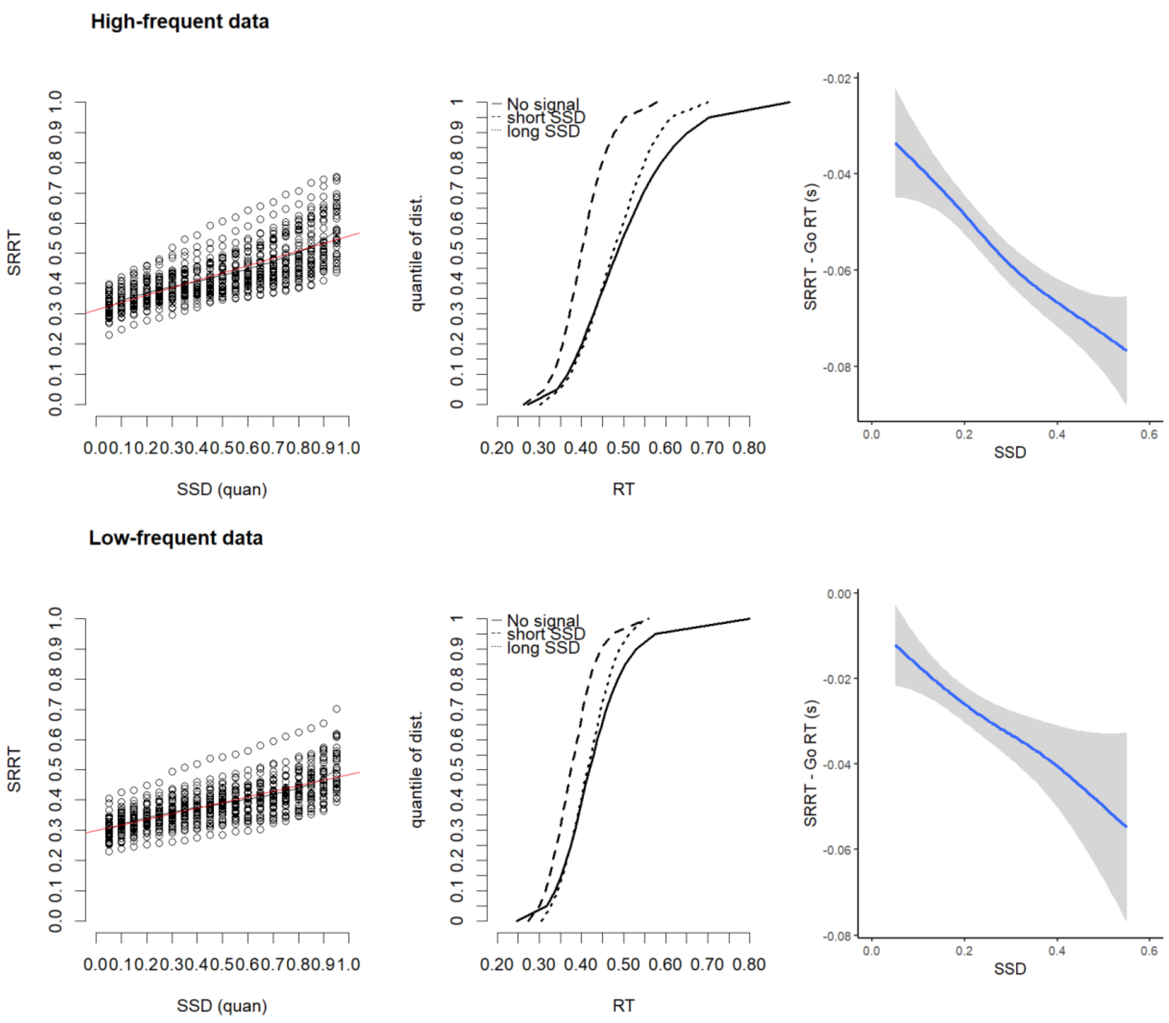

Figure C. *Tests of context independence violations in Experiment 1. (left) Mean SRRTs over quantiles of SSDs (plus a fitted regression line in red); violations would be indicated as any SRRT at SSD quantile n being lower than the SRRT at quantile n-1. (middle) Cumulative density distributions of Go RT on go trials; SRRT on short-SSD stop trials [i.e., SSD < mean (SSD)], or SRRT on long-SSD stop trials [i.e., SSD > mean (SSD)]; violations would be indicated as (1) the functions not having a common minimum RT and/or (2) the steepness of the function not progressing from Go RT (least steep), SRRT on long-SSD stop trials, to SRRT on short-SSD stop trials (steepest).*[1] *(right) Check as described by Bissett et al. (2021):*

[1] The tails are predicted by the horse-race model to be particularly long for short-SSD trials, which is not what we observed here.”.

the SRRT minus a matched Go RT; violations would be indicated as values larger than 0.[2] *None of the violations were observed.*

[2] The horse-race model additionally predicts that the difference between SRRT and matched Go RT decreases over SSD, which is not what we observed here. This pattern of results is in line, however, with Bissett et al. (2021)'s findings on context-independence violations.

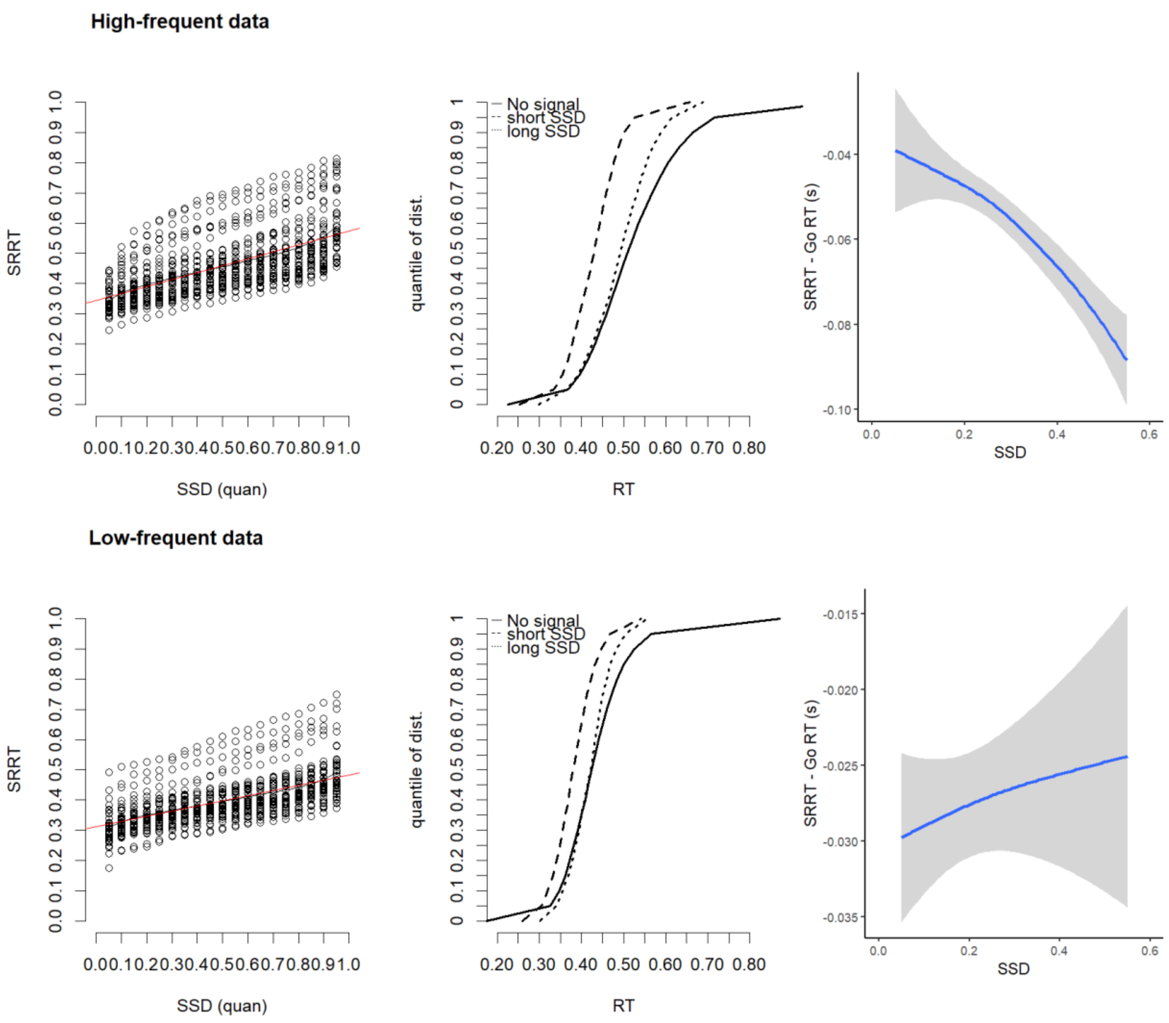

Figure D. *Tests of context independence violations in Experiment 2. See Appendix-Figure C for more details. Again, none of the violations were observed, although the positive trend of the low-frequent SRRT minus matched Go RT function was not expected (see subplot C-top).*
